# Supervised and Unsupervised Learning with Numerical Computation for the Wolfram Cellular Automata

**DOI:** 10.3390/e27111155

**Published:** 2025-11-14

**Authors:** Kui Tuo, Shengfeng Deng, Yuxiang Yang, Yanyang Wang, Qiuping Wang, Wei Li, Wenjun Zhang

**Affiliations:** 1 Key Laboratory of Quark and Lepton Physics (MOE) and Institute of Particle Physics, Central China Normal University, Wuhan 430079, China; tuokui@mails.ccnu.edu.cn (K.T.); yuxyang@mails.ccnu.edu.cn (Y.Y.); yanyangwang@mails.ccnu.edu.cn (Y.W.); 2 School of Physics and Information Technology, Shaanxi Normal University, Xi’an 710061, China; dengsf@snnu.edu.cn; 3 Laboratoire Systèmes Complexes et Information Quantique (SCIQ) Lab, ESIEA, 74 bis Avenue Maurice Thorez, 94200 Ivry sur Seine, France; alexandre.wang@esiea.fr; 4 School of Medical Information Engineering, Anhui University of Chinese Medicine, Hefei 230012, China

**Keywords:** Wolfram cellular automata, numerical computation, supervised learning, unsupervised learning, asymptotic density

## Abstract

The local rules of elementary cellular automata (ECA) with one-dimensional three-cell neighborhoods are represented by eight-bit binary numbers that encode deterministic update rules. This class of systems is also commonly referred to as the Wolfram cellular automata. These automata are widely utilized to investigate self-organization phenomena and the dynamics of complex systems. In this work, we employ numerical simulations and computational methods to investigate the asymptotic density and dynamical evolution mechanisms in Wolfram automata. We explore alternative initial conditions under which certain Wolfram rules generate similar fractal patterns over time, even when starting from a single active site. Our results reveal the relationship between the asymptotic density and the initial density of selected rules. Furthermore, we apply both supervised and unsupervised learning methods to identify the configurations associated with different Wolfram rules. The supervised learning methods effectively identify the configurations of various Wolfram rules, while unsupervised methods like principal component analysis and autoencoders can approximately cluster configurations of different Wolfram rules into distinct groups, yielding results that align well with simulated density outputs. Machine learning methods offer significant advantages in identifying different Wolfram rules, as they can effectively distinguish highly similar configurations that are challenging to differentiate manually.

## 1. Introduction

Cellular automata (CA) are dynamical systems discrete in time, space, and state. The Game of Life [1] represents a canonical cellular automaton that investigates how simple rules evolve within discrete spaces and whether such rules can generate dynamics simulating lifelike phenomena. However, its limitation lies in its reliance on a single fixed rule set. In contrast, Wolfram cellular automata enable the exploration of emergent complexities across diverse deterministic rules [2], revealing how intricate phenomena arise from elementary local interactions. While Wolfram automata operate under deterministic constraints, the Domany-Kinzel (DK) model generalizes this framework as a stochastic cellular automaton [3], facilitating the study of non-equilibrium dynamics and phase transitions in disordered systems. Cellular automata are widely used in various fields, such as fluid mechanics [4], ecosystem modeling [5], simulation of any computer algorithm [6], urban planning [7], simulating traffic flow [8], mathematics [9], stock market [10], crystal growth models [11], and biological modeling [12].

The intricate patterns generated by CA present formidable challenges for conventional analytical methods, motivating the adoption of advanced computational approaches. Machine learning approaches have garnered significant interest in recent years and have been extensively adopted across diverse domains, such as computer vision [13,14], natural language processing [15], recommendation systems [16], finance [17], healthcare [18,19], and large language models (LLMs) [20].

Machine learning, leveraging its robust capabilities in high-dimensional data processing and nonlinear modeling, has been extensively applied to research in physics. For example, in high-energy physics, particle swarm optimization and genetic algorithms have been employed to autonomously optimize hyperparameters of machine learning classifiers in high-energy physics data analyses [21]; HMPNet integrates HaarPooling with graph neural networks to boost quark–gluon tagging accuracy [22]. In astrophysics, a text-mining-based scientometric analysis has been conducted to map the application trends of machine learning in astronomy [23], and a machine learning model has been developed to predict cosmological parameters from galaxy cluster properties [24]. In quantum simulation, an ab initio machine learning protocol has been developed for intelligent certification of quantum simulators [25], a quantum machine learning framework has been developed for resource-efficient dynamical simulation with provable generalization guarantees [26], and a noise-aware machine learning framework has been developed for robust quantum entanglement distillation and state discrimination over noisy classical channels [27].

Machine learning has enabled groundbreaking advances in identifying distinct phases of matter. Carrasquilla and Melko’s seminal 2016 study demonstrated that supervised machine learning methods can classify ferromagnetic and paramagnetic phases in the classical Ising model [28], accurately extracting critical points and spatial correlation exponents. This pioneering work catalyzed the widespread adoption of machine learning for analyzing diverse phase transitions across condensed matter systems. Machine learning has also been applied to identify more complex phase transitions, such as in three-dimensional Ising models [29], percolation phase transitions [30], topological phase transitions [31,32], the non-equilibrium phase transitions in the Domany-Kinzel automata [33], and the non-equilibrium phase transitions in even-offspring branching annihilating random walks [34].

In this work, we study the (1+1)-dimensional Wolfram automata by using numerical computation and machine learning. Wolfram automata encompass diverse evolution rules, enabling in-depth analysis of dynamical evolution mechanisms in cellular systems. Simulations and numerical computations are employed to investigate the fractal structures, transient time to reach steady states, and asymptotic density of Wolfram automata. Manual analysis struggles to distinguish configurations under complex Wolfram rules, whereas machine learning leverages its image-classification capabilities to automate Wolfram automata identification. By applying both supervised and unsupervised learning methods, a trained neural network can accurately identify diverse configurations across different Wolfram rules after training on a limited set of automata samples. In addition, two unsupervised learning methods, principal component analysis (PCA) and autoencoders, are utilized to classify configurations and estimate the density of Wolfram automata.

The remainder of this paper is organized as follows: In Section 2, we briefly introduce the Wolfram automata. Section 3 presents the numerical computations of (1+1)-dimensional Wolfram automata, which are employed to investigate the fractal structures and asymptotic density of Wolfram automata. Section 4 presents the supervised learning of (1+1)-dimensional Wolfram automata, which are used to identify distinct configurations of Wolfram rules. Section 5 is about the unsupervised learning results of (1+1)-dimensional Wolfram automata, via autoencoder and PCA. Section 6 summarizes the main findings of this work.

## 2. Wolfram Cellular Automata

Wolfram cellular automata operate based on evolution rules governed by deterministic dynamics, as noted in prior studies [2]. Despite their simplicity, these rules can generate complex phenomena. In one-dimensional Wolfram automata, each site (or cell) assumes a binary state (0 or 1). Formally, such systems are described as (1+1)-dimensional cellular automata, where the first dimension represents discrete spatial extent and the second corresponds to discrete temporal evolution. When restricted to nearest-neighbor interactions, the neighborhood of a cell comprises itself and its immediate left and right neighbors, forming cellular automata with a three-cell neighborhood. This type of automaton is referred to as an elementary cellular automaton.

The local rules governing one-dimensional cellular automata with a three-cell neighborhood can be fully specified by an 8-bit binary number. Each such binary number corresponds to a unique rule identifier expressed as a decimal value between 0 and 255, resulting in 256 distinct possible rules. This arises because the next state of a cell depends on its own state and those of its two immediate neighbors—a three-cell configuration with two possible states per cell, yielding $2^{3}$ neighborhood patterns. Each pattern maps to one of two possible output states, leading to $2^{2^{3}}$ = 256 total rules.

**Figure 1:**
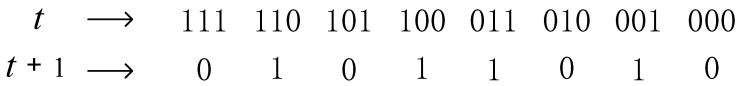
An example of the local rule for the time evolution of one-dimensional Wolfram automaton. The numerical representation of rule 90. According to Equation (1), this gives rule 90.

The output values $a_{i}$ for each input condition of the Wolfram automaton are arranged in descending order of the corresponding neighborhood state index (i.e., $a_{7}$ to $a_{0}$), forming an 8-bit binary number. The rule number *R* is computed as the decimal equivalent of this binary representation:

(1) $$R=\sum_{i=0}^{7}2^{i}a_{i},$$

where $a_{i}$ denotes the output state for the *i*-th neighborhood configuration. For example, Figure 1 shows the numerical representation of Wolfram rule 90 (hereinafter rule 90), where the binary sequence 01011010 yields $R=2^{6}+2^{4}+2^{3}+2^{1}=90$.

The density of the Wolfram automata is defined as the ensemble average of the fraction of active sites (sites with a value of 1) at time step *t*:

(2) $$\rho \left(t\right)=\langle \frac{1}{L}\sum_{i}s_{i}\left(t\right)\rangle \;,$$

where *L* is the lattice size, $s_{i}\left(t\right)\in \left\{0,1\right\}$ is the state of site *i* at time step *t*, and $\langle \cdot \rangle$ denotes averaging over independent initial conditions. After the long-time evolution, ρ(t) converges to a stationary value $\rho_{stat}$.

The asymptotic density $\rho_{\infty }$ is defined by the following:

(3) $$\rho_{\infty }=\lim_{t\to \infty }\lim_{L\to \infty }E\left[\rho_{L}\left(t\right)\right],$$

Here, $\rho_{L}\left(t\right)$ denotes the density at time step *t* for a system of size *L*, and E[·] represents the expectation operator. The order of limits is critical: first, the system size *L* is taken to infinity to remove boundary constraints and finite-size effects; then, time step *t* is extended to infinity to capture the steady-state behavior. The term “Monte Carlo” in this context specifically refers to sampling over independent and identically distributed (i.i.d.) initial configurations of the Wolfram automata, where each simulation starts from randomly generated initial conditions. This differs from Markov-chain Monte Carlo (MCMC) methods, which rely on sequential correlated samples.

## 3. Numerical Computation of Wolfram Automata

We first employ Monte Carlo simulation methods to study the Wolfram automata, leveraging randomly generated initial configurations to analyze fractal structures and self-organization phenomena. This approach specifically investigates the relationship between the asymptotic density of evolved states and the disordered initial state of the automaton. Subsequently, we utilize numerical computation to explore the evolution mechanisms of cellular automata configurations under predefined Wolfram rules.

**Figure 2:**
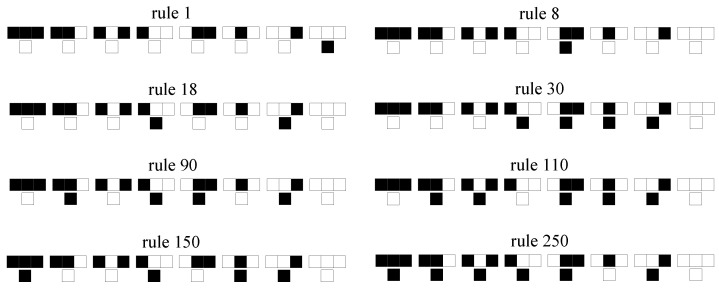
A black-and-white representation of the Wolfram rule. Black represents sites with value 1 and white represents empty sites.

The local rules governing Wolfram automata can be formalized as Boolean functions acting on a central site and its nearest neighbors. Figure 2 shows a black-and-white color representation of the Wolfram rule, where black represents sites with value 1 and white represents sites with value 0. Each rule is defined by a unique Boolean expression, as shown in Table 1. For example, rule 90 is equivalent to $s_{i}\left(t+1\right)$ = $s_{i-1}\left(t\right)⊕s_{i+1}\left(t\right)$, rule 18 to $s_{i}\left(t+1\right)$ = $\neg s_{i}\left(t\right)\wedge \left(s_{i-1}\left(t\right)⊕s_{i+1}\left(t\right)\right)$, rule 30 to $s_{i}\left(t+1\right)$ = $s_{i-1}\left(t\right)⊕\left(s_{i}\left(t\right)\vee s_{i+1}\left(t\right)\right)$, and 110 to $s_{i}\left(t+1\right)$ = $\left(s_{i}\left(t\right)\wedge \left(\neg s_{i-1}\left(t\right)\right)\right)\vee \left(s_{i}\left(t\right)⊕s_{i+1}\left(t\right)\right)$. Here, $s_{i}\left(t\right)$ denotes the state of site *i* at time step *t*, while $s_{i-1}\left(t\right)$ and $s_{i+1}\left(t\right)$ represent its left and right neighbors, respectively. Boolean operators include AND (∧), OR (∨), NOT (¬), and XOR (⊕). Compared to enumerating all 256 rule conditions, these simplified Boolean expressions significantly enhance computational efficiency in simulations by reducing logical redundancy and enabling optimized code implementation.

**Figure 3:**
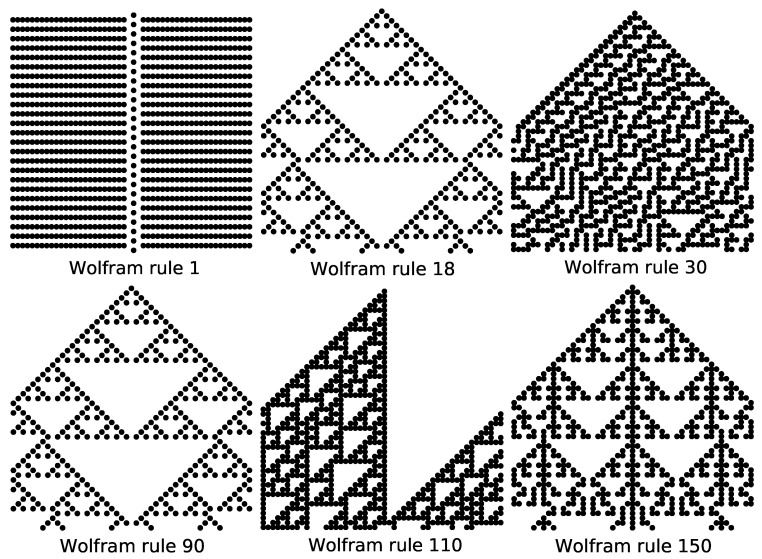
Evolution of the configurations of the Wolfram automaton under the initial state with a single active site. Size L=50; time steps t=50.

As shown in Figure 3, the cluster diagram of the Wolfram automata evolves from an initial state with a single active site. Starting from such simple initial configurations, Wolfram automata either evolve to homogeneous states or generate self-similar fractal patterns. For example, under the initial state with a single active site, rules 18 and 90 both produce the Sierpiński triangle pattern with a fractal dimension of $\log_{2}3\approx 1.585$. Similarly, rule 150 exhibits self-similarity with a fractal dimension of 1.69 [35].

Numerical analysis reveals that the emergent Sierpiński triangle structure in rules 18 and 90 depends solely on two specific rule conditions: neighborhood state 100→1 and 001→1. Notably, rule 90 additionally satisfies conditions such as 110→1 and 011→1, but these do not influence the Sierpiński pattern under the specified initial state. This property extends to other rules satisfying the same core conditions (100→1 and 001→1), resulting in eight distinct Wolfram rules (including rules 26, 82, 146, 154, 210, and 218) that generate Sierpiński triangles when evolved from configurations containing only one active site at any time step.

In Ref. [36], the explanation of symmetry originates from the additive nature of cellular automata rules. Rules 150 and 105 exhibit unique symmetry because they are additive rules, which flip the result only when any of the three-cell neighborhood states is flipped, leading to the emergence of self-similar fractal structures like the Sierpiński triangle. Similarly, rule 90 and its equivalence class, {18, 22, 26, 82, 146, 154, 210, and 218}, also generate the Sierpiński triangle from a single active site through their additive properties, forming a symmetric octet. These rules are equivalent under transformation, reflecting an underlying symmetry.

**Figure 4:**
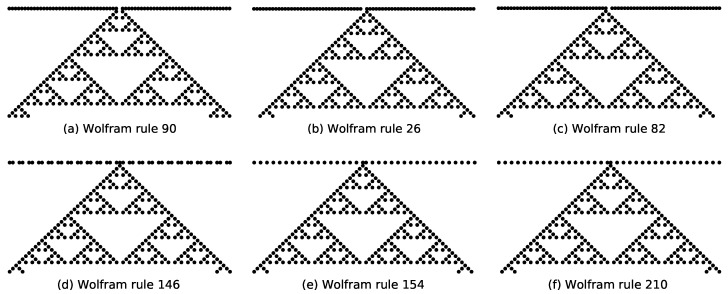
Evolution of the configurations of (**a**) rule 90, (**b**) rule 26, and (**c**) rule 82 under an initial state where the central site is 0 and all other sites are 1. Evolution of the configurations of (**d**) rule 146 under the initial state “…0, 1, 1, 0, 1, 1, 0, 1, 1, 1, 0, 1, 1, 0, 1, 1…”, (**e**) rule 154 under the initial state “…0, 1, 0, 1, 0, 1, 1, 0, 1, 0, 1…”, and (**f**) rule 210 under the initial state “…0, 1, 0, 1, 0, 1, 1, 0, 1, 0, 1…”.

Further investigations under modified initial conditions highlight nuanced behavioral differences. For rule 90 evolving from an initial state where the central site is 0 and all others are 1, the state transition at t=1 depends exclusively on 110→1 and 011→1. This results in the activation of two central sites at this time step and ultimately forms a Sierpiński triangle with vertices removed (Figure 4a). Under identical initial conditions, the transition of rule 26 at t=1 depends solely on 011→1, while rule 82 depends solely on 110→1. Both rules yield only one active site at this time step, and yet subsequently evolve into full Sierpiński triangles (Figure 4b,c). For specialized initial states (e.g., alternating sequences with isolated active sites), rules 146, 154, and 210 each activate only one site at t=1 due to distinct rule dependencies, (111→1 for rule 146, 011→1 for rule 154, and 110→1 for rule 210), yet all subsequently generate complete Sierpiński triangles (Figure 4d,f).

**Figure 5:**
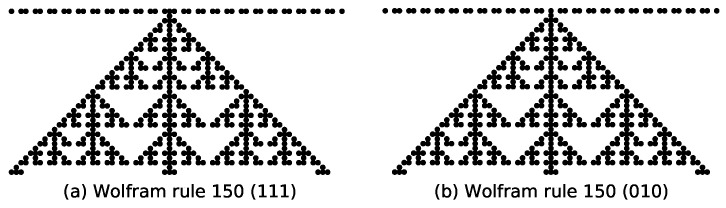
Evolution of the configurations of (**a**) rule 150 under the initial state “…1, 1, 0, 1, 1, 0, **1, 1, 1,** 0, 1, 1, 0, 1, 1…”, and (**b**) rule 150 under the initial state “…1, 1, 0, 1, 1**, 0, 1, 0**, 1, 1, 0, 1, 1…”.

Under the initial state with a single active site, rule 150 generates a self-similar pattern with a fractal dimension of 1.69 [2]. Further numerical analysis reveals that if a configuration at any time step *t* contains exactly one active site, rule 150’s subsequent evolution will consistently produce self-similar patterns with the same fractal dimension of 1.69. This property holds across diverse initial states; for the initial state “…1, 1, 0, 1, 1, 0, 1, 1, 1, 0, 1, 1, 0, 1, 1…”, the state transition at t=1 depends solely on the rule condition 111→1, resulting in a single active site; and for the initial state “…1, 1, 0, 1, 1, 0, 1, 0, 1, 1, 0, 1, 1…”, the transition at t=1 relies exclusively on 010→1, also yielding one active site. Despite these distinct initial configurations and localized rule dependencies, rule 150’s long-term evolution converges to identical self-similar structures with fractal dimension 1.69, as shown in Figure 5.

Grassberger and Wolfram conducted theoretical analyses primarily focused on complex Wolfram rules [2,37], such as rules 90 and 182, which exhibit well-defined asymptotic density $\rho_{\infty }$. This asymptotic density is typically independent of the initial state density. However, under specific initial conditions, for example, a configuration with only a single active site, rule 90 generates Sierpiński triangle patterns. This implies that its asymptotic density displays periodic oscillations rather than converging to a constant value. The observed divergence from their established $\rho_{\infty }$ motivates further investigation into whether the asymptotic behavior of complex Wolfram rules under very low initial densities aligns with their behavior under disordered initial states. Notably, the steady-state density of many Wolfram rules are known to depend on initial conditions, which prompts a systematic exploration of the relationship between initial density and long-term statistical behavior. In this work, we employ numerical simulation and analysis to study the temporal evolution of configurations in these automata, aiming to characterize their dynamic properties.

**Figure 6:**
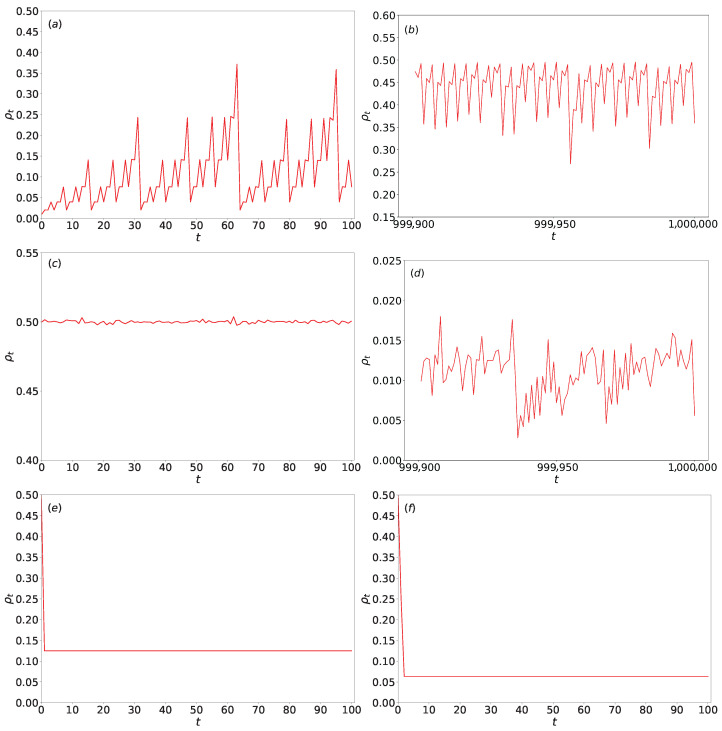
The temporal evolution of density $\rho_{t}$ for various Wolfram rules under different initial densities. (**a**) $\rho_{t}$ for rule 90 with initial density $\rho_{0}$ = 0.01, system size *L* = 2000, and simulation steps *t* = 100. (**b**) The same rule 90 system under identical initial conditions, but during the last 100 steps of an extended simulation ($10^{6}$). (**c**) Rule 90 with $\rho_{0}$ = 0.5, *L* = 1000, and *t* = 100. (**d**) Rule 18 with $\rho_{0}$ = 0.99, *L* = 2000, observed during the last 100 steps of $10^{6}$. (**e**) Rule 2 with $\rho_{0}$ = 0.5, *L* = 1000, and *t* = 100. (**f**) Rule 36 under identical initial conditions to rule 2. For each panel, $\rho_{t}$ is computed by averaging over 100 independent initial density configurations at each time step.

Monte Carlo simulations of (1+1)-dimensional Wolfram automata are conducted according to predefined Wolfram rules under periodic boundary conditions. When starting from a disordered initial state where each site has an independent probability *p*, the initial density $\rho_{0}$ equals *p*. Figure 6 illustrates the temporal evolution of density $\rho_{t}$ for various Wolfram rules under different initial densities. We selected specific rules including rules 90, 18, 2, and 36 for presentation. These rules exemplify distinct behavioral types within Wolfram automata: rules 90 and 18 are complex rules demonstrating chaotic and fractal characteristics as simple rules that serve as discriminative filters for selected initial configurations, while rules 2 and 36 rapidly converge to stable states. This selection is designed to contrast the differences in density evolution across rules of varying complexity, thereby emphasizing our research focus.

As shown in Figure 6a,b, for rule 90 with a very low initial density ($\rho_{0}$ = 0.01, 2000), the density $\rho_{t}$ exhibits significant fluctuations over the first *t* = 100 time steps. During the last 100 steps of a prolonged simulation (*t* = $10^{6}$), $\rho_{t}$ also exhibits significant fluctuations with distinct periodic characteristics. Due to large fluctuations, temporal moving averages are adopted here. With ensemble averaging over 100 independent initial condition seeds, ρ approximately equal 0.46, which is lower than the theoretical asymptotic density $\rho_{\infty }=0.5$. In contrast, for rule 90 with a moderate initial density ($\rho_{0}$ = 0.5, 2000, *t* = 100), as seen in Figure 6c, $\rho_{t}$ is the absence of significant fluctuations beyond early time. $\rho_{t}$ converges rapidly to the asymptotic value of 0.5 within 600 time steps and remains stable.

For rule 18 with a very high initial density ($\rho_{0}$ = 0.99, *L* = 2000), Figure 6d shows that during the last 100 steps of $10^{6}$ steps simulation, $\rho_{t}$ exhibits minor fluctuations and remains small.

In the cases of rules 2 and 36 with initial density $\rho_{0}$ = 0.5 (Figure 6e,f), both rules achieve stability quickly: rule 2 stabilizes almost immediately, while rule 36 converges within a few time steps.

A key observation is that for complex rules like rule 90, under typical initial densities (e.g., $\rho_{0}$ = 0.5), $\rho_{t}$ stabilizes within a few hundred steps. However, under extreme initial densities (very low or very high), like *L* = 2000, and even prolonged evolution (e.g., *t* = $10^{6}$), $\rho_{t}$ is lower than the theoretical asymptotic density $\rho_{\infty }=0.5$. This discrepancy may stem from finite-size effects or insufficient time steps (although a large $t=10^{6}$) in numerical simulations. As the system size increases, $\rho_{t}$ will approach the asymptotic density more closely; a more detailed discussion is provided in the subsequent section. In contrast, simple rules (such as rules 2 and 36) exhibit rapid convergence and stable densities regardless of initial conditions.

**Figure 7:**
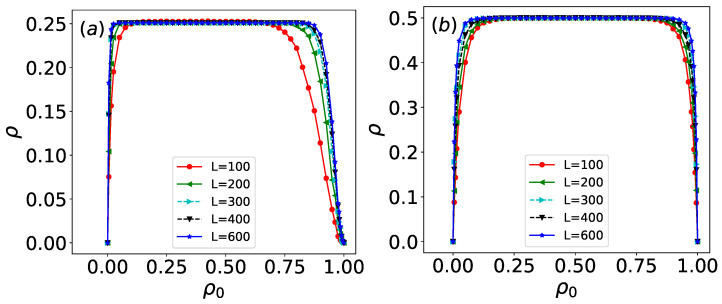
Density ρ of (**a**) rule 18 and (**b**) rule 90 under long-time evolution for disordered initial states with density $\rho_{0}$ where *L* = 100, 200, 300, 400, 600; The corresponding time step *t* = 6000.

For rules 18 and 90, simulations are run on arrays of sizes *L* = 100, 200, 300, 400, and 600, and time step *t* = 6000, with the configurations from the last 200 time steps (Δt = 200) being taken. For each initial density $\rho_{0}$, 1000 configurations are generated to calculate the density. As shown in Figure 7, the long-time evolved density of both rules under disordered initial states is presented. For most initial densities $\rho_{0}$, rules 18 and 90 exhibit well-defined asymptotic densities: $\rho_{\infty }=0.25\pm 0.002$ for rule 18 and $\rho_{\infty }=0.5\pm 0.002$ for rule 90. However, under extreme initial densities (e.g., $\rho_{0}\leq 0.01$ or $\rho_{0}\geq 0.99$), the evolved density significantly deviates from these asymptotic values.

At extremely low initial densities (characterized by very few active sites), the evolution of both rules depends primarily on the transition conditions 100→1 and 001→1. In this regime, sites evolve nearly independently. When only a single site is active in the initial state, both rules generate Sierpiński triangle patterns, resulting in densities much lower than their asymptotic values. Under such sparse initial conditions, the evolution produces numerous isolated triangular structures with large empty regions, leading to low overall density. As the initial density increases, the evolved density converges toward the asymptotic value. Conversely, at extremely high initial densities, the first time step is dominated by the condition 111→0, causing a sharp drop in density. This results in configurations similar to those observed under very low initial densities. As the initial density decreases from this extreme, the evolved density again approaches the asymptotic value. For a fixed initial density, larger system sizes (increasing *L*) yield densities closer to the asymptotic limit.

**Figure 8:**
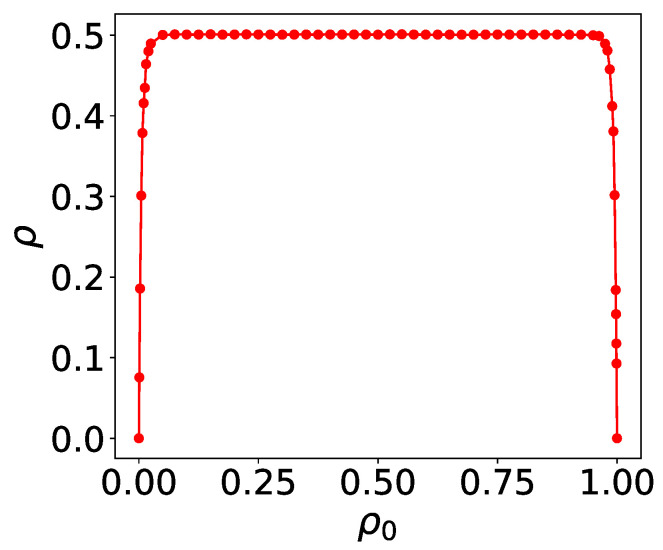
Density ρ of rule 126 under long-time evolution for disordered initial states with density $\rho_{0}$. *L* = 200, *t* = 6000.

Rule 126, another complex rule, is simulated on arrays of size *L* = 200 and *t* = 6000, with the configurations from the last 200 time steps (Δt = 200) being taken. For each initial density $\rho_{0}$, 1000 configurations are generated to calculate the density. As shown in Figure 8, the long-time density ρ for disordered initial states is presented. Unlike rules 18 and 90, rule 126 exhibits a consistent asymptotic density $\rho_{\infty }=0.5\pm 0.002$ across a broader range of initial densities. This result holds robustly under varying initial densities and system sizes.

**Figure 9:**
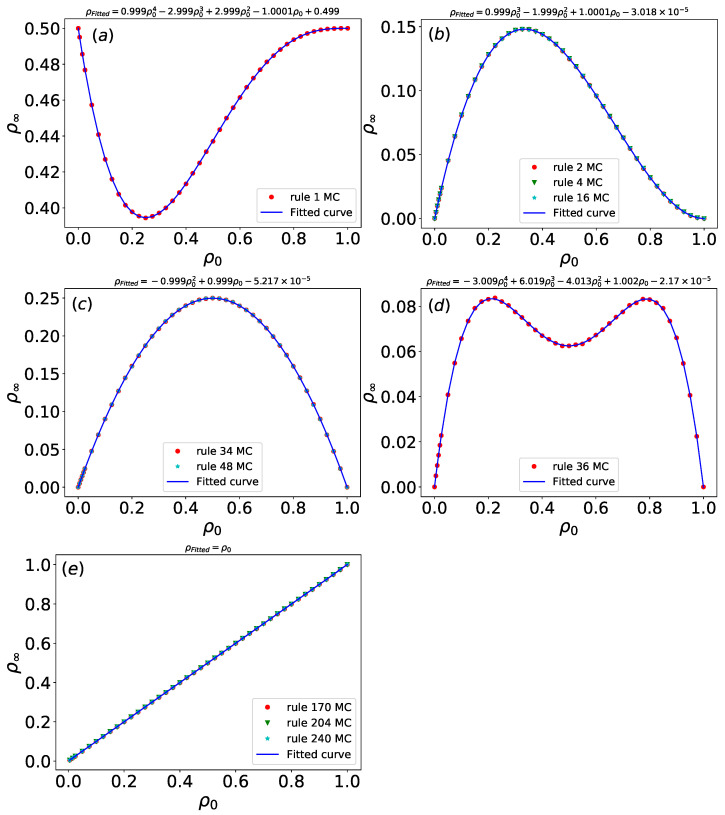
Asymptotic density $\rho_{\infty }$ of Wolfram rules under disordered initial configurations with varying $\rho_{0}$, and fitting results for $\rho_{\infty }$. (**a**) rule 1; (**b**) rules 2, 4, and 16; (**c**) rules 34 and 48; (**d**) rule 36; (**e**) rules 170, 204, and 240. *L* = 1000; *t* = 30. Red dots represent asymptotic density of Wolfram rules and blue lines represent the fitting curve.

For rules 1, 2, 4, 16, 34, 48, 36, 170, 204, and 240, simulations are run on arrays of size *L* = 1000, and time steps *t* = 30, with the configurations from the last time step being taken. For each initial density $\rho_{0}$, 1000 configurations are generated to calculate the density. The density ρ of these simple Wolfram rules stabilizes rapidly, with the asymptotic density $\rho_{\infty }$ exhibiting dependence on the initial density $\rho_{0}$. As shown in Figure 9, the asymptotic density $\rho_{\infty }$ under disordered initial configurations with varying $\rho_{0}$ is presented for all rules, including parameter optimization and fitting results. A least squares fitting procedure yields fitted densities $\rho_{Fitted}$ for each rule, and approximate values of $\rho_{\infty }$ are summarized in Table 2.

For rule 1, whose evolution depends solely on the condition 000→1, the asymptotic density $\rho_{\infty }$ = 0.5 when $\rho_{0}$ is 0 or 1. Conventional analytical methods struggle to determine the extrema of such functions, but our approach employs Python 3.10’s symbolic computation library (SymPy) to obtain precise solutions. Specifically, a minimum density of 0.395 occurs at $\rho_{0}=0.25$.

The asymptotic density curves for rules 2, 4, and 16 coincide apart from minor fluctuations. A maximum density of 0.148 is at $\rho_{0}=0.333$. The evolution of rule 2 depends only on 001→1, rule 4 on 010→1, and rule 16 on 100→1. Despite their distinct evolutionary paths under the same initial density, all three rules converge to the same asymptotic density, indicating functionally equivalent dynamical mechanisms. This suggests that these rules can serve as effective filters in certain applications.

Similarly, rules 34 and 48 exhibit identical asymptotic density $\rho_{\infty }$. Rule 34 depends on condition 101→1 and 001→1, while rule 48 depends on condition 101→1 and 100→1. At $\rho_{0}=0.5$, both rules reach a maximum density of 0.25.

For rule 36, the fitted density curve displays two local maxima and one local minimum. The asymptotic density reaches a local minimum of 0.0625 at $\rho_{0}=0.5$, and local maxima of approximately 0.0833 occur at $\rho_{0}=0.211$ or $\rho_{0}=0.789$.

Rules 170, 204, and 240 all satisfy $\rho_{\infty }=\rho_{0}$. Rule 204 is the “identity rule”, defined by the Boolean function $s_{i}\left(t+1\right)=s_{i}\left(t\right)$, meaning the configuration remains unchanged over time. Rule 170 is characterized by $s_{i}\left(t+1\right)=s_{i+1}\left(t\right)$, and rule 240 by $s_{i}\left(t+1\right)=s_{i-1}\left(t\right)$. The asymptotic density of these rules always matches the initial density, confirming that they share identical dynamical evolution mechanisms.

Simple Wolfram rules such as rules 2, 4, 16, 34, and 48 stabilize rapidly within a small number of time steps, with the asymptotic density $\rho_{\infty }$ depending on the initial density $\rho_{0}$. Notably, despite being governed by distinct local update conditions, for instance, rule 2 depends on 001→1, rule 4 on 010→1, and rule 16 on 100→1. These rules exhibit identical asymptotic densities under the same initial density $\rho_{0}$, even though their configurations evolve through different pathways over time. This convergence behavior suggests that they share equivalent underlying dynamical mechanisms, irrespective of differences in their local transition functions.

## 4. Supervised Learning of the Wolfram Automata

Supervised learning is applied to study Wolfram automata, with data comprising raw configurations generated from Monte Carlo (MC) simulations of the (1+1)-dimensional Wolfram automata. Within the (1+1)-dimensional Wolfram automata framework encompassing 256 distinct rules, certain rules rapidly converge to trivial steady states (e.g., homogeneous or empty configurations), enabling straightforward classification. We focus on Wolfram rules that exhibit complex structural configurations under disordered initial states during temporal evolution. Ten typical Wolfram rules are selected: rules 6, 16, 18, 22, 36, 48, 90, 150, 182, and 190. The generated configurations are divided into a training set and a test set. the configurations $x_{i}$ of each rule are labeled with an identical label. For ten distinct Wolfram rules, the true label $y_{i}$ is a **one-hot vector**. For instance, rule 6 corresponds to [1,0,0,…], and rule 150 corresponds to […,0,1,0,0], where only the position indexing the specific rule is set to 1, and all others are 0.

**Figure 10:**
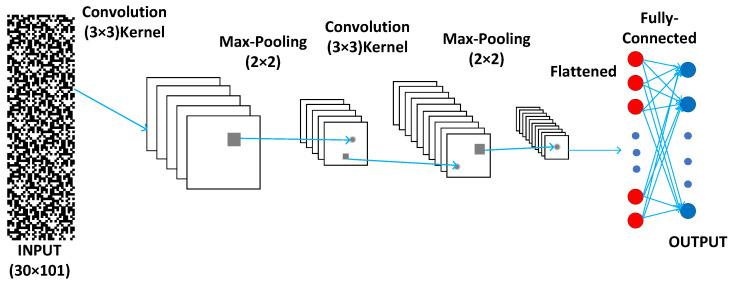
The CNN architecture consists of two convolutional layers, each followed by a max-pooling layer, a fully connected layer, and a **softmax** output layer. The first convolutional layer applies 3×3 **kernels** with **sigmoid** activation to extract spatial features, followed by 2×2 max-pooling for dimensionality reduction. The second convolutional layer repeats this pattern, further refining feature maps. The pooled features are then flattened and passed through a fully connected layer using **sigmoid** activation. Finally, the output layer employs **softmax** activation to produce class probabilities for classification tasks.

For supervised learning, we apply the convolutional neural network (CNN) as illustrated in Figure 10. The CNN architecture consists of two convolutional layers, each followed by a max-pooling layer, a fully connected layer, and a **softmax** output layer. The first convolutional layer applies 3 × 3 **kernels** with **sigmoid** activation, extracting spatial features while introducing nonlinearity, followed by 2 × 2 max-pooling for dimensionality reduction, enhancing translational invariance. The second convolutional layer repeats this pattern, further refining feature maps. The pooled features are then flattened and passed through a fully connected layer using **sigmoid** activation. Finally, the output layer employs **softmax** activation to produce class probabilities for classification tasks.

Since learning machines extract features from configuration images generated across distinct rules and small system sizes, selecting configurations that maximize information capture is essential. For complex Wolfram automata such as rules 18 and 90, under low or high initial densities (e.g., $\rho_{0}\leq 0.01$ or $\rho_{0}\geq 0.99$), the density evolving over time remains low; thus, it is customary to select random initial states (probability of one per site is 0.5) for simulations to capture non-trivial dynamical behaviors. For simple rules, the asymptotic density $\rho_{\infty }$ depends on the initial density. Therefore, the initial density $\rho_{0}$ corresponding to a high asymptotic density should be optimally selected. For example, rule 16 adopts an initial state density $\rho_{0}$ of 0.33, while rule 36 sets it to 0.21.

The configuration images are of L×(t+1) dimension, L=30, and t=100. For each rule, 2000 labeled configurations are generated for the training set and another 200 configurations for the test set. The CNN output layers are eventually averaged over the test set.

Categorical cross-entropy loss is the standard choice for data classification tasks in machine learning. For Wolfram rule classification, it thus serves as the designated loss function. The Categorical cross-entropy loss function is defined as follows:

(4) $$L=-\frac{1}{N}\sum_{i=1}^{N}\log\left({\hat{y}}_{i,k_{i}}\right),$$

*N* is the number of samples in the batch (batch size), and ${\hat{y}}_{i,k_{i}}$ is the model’s predicted probability for the true class label $k_{i}$ of sample *i*.

**Figure 11:**
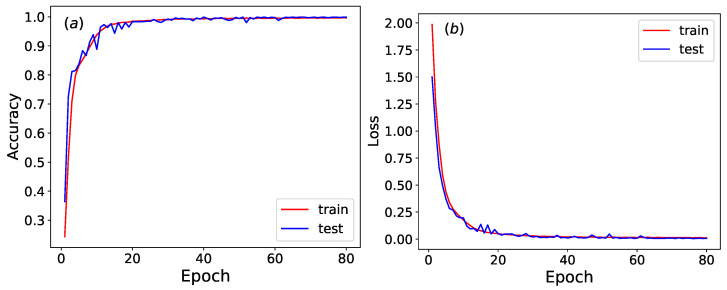
(**a**) The accuracy and (**b**) loss function of the trained neural network across epochs.

As illustrated in Figure 11, the accuracy and loss function of the trained neural network stabilize around epoch 80. The CNN achieves an impressive 99.9% test accuracy in classifying Wolfram rules. This high level of recognition accuracy highlights the CNN’s exceptional ability to reliably classify Wolfram rules, even within complex systems.

A confusion matrix is a tabular tool used to evaluate the performance of a classification model, in which the rows represent the true classes (actual values), the columns represent the predicted classes (model outputs), the diagonal entries indicate the number of correctly classified samples, and the off-diagonal entries reveal specific patterns of misclassification. This structure helps quickly identify the accuracy of the model across different classes and trends in errors. As shown in Table 3, this is a confusion matrix for the CNN-based classification of Wolfram rules, with identical initial density conditions in both the training and test sets. The model demonstrates excellent classification accuracy, with nearly perfect diagonal values (200 correct predictions for most rules) and only three misclassifications out of 2000 samples.

A classification report is a concise summary that evaluates the performance of a classification model by presenting key metrics such as precision, recall, and F1-score. Precision is the proportion of correctly predicted positive cases (e.g., rule 6) out of all cases predicted as positive. High precision (close to 1.0) indicates few false positives. Recall is the proportion of correctly predicted positive cases out of all actual positive cases. High recall (close to 1.0) indicates few false negatives. F1-score is the harmonic mean of precision and recall, providing a balanced measure between them. It is particularly useful when class distribution is uneven (though here all classes have equal support). Class refers to the Wolfram automata representing different rules. As shown in Table 4, this is a classification report of CNN of Wolfram rules, with identical initial density conditions in training and test sets. The classification report demonstrates exceptional performance of the CNN model in classifying Wolfram automata rules.

In order to verify the robustness of the neural network in recognizing Wolfram automata, test configurations with initial densities different from those used in the training set are input into a pre-trained CNN. This process evaluates the accuracy of classifying distinct rules. The initial densities are uniformly sampled from the values 0.2, 0.4, 0.6, and 0.8, with 200 test samples generated for each rule. As shown in Table 5 and Table 6, the provided confusion matrix and classification report collectively evaluate the CNN model’s performance on Wolfram rules classification under different initial density conditions between training and test sets. The confusion matrix indicates an overall accuracy of approximately 89.65% (1793 correct predictions out of 2000), with notable misclassification patterns, such as rule 16 being frequently confused with rules 18 (41 errors) and 48 (19 errors). Correspondingly, the classification report reveals varied metric scores, where rules 16 and 36 show lower F1-scores (0.748 and 0.778, respectively) due to reduced recall or precision, while rules 182 and 190 achieve perfect scores.

Compared to scenarios with identical initial density conditions in training and test sets, it is observed that when test densities differ from those in the training set, the accuracy of classifying distinct rules declines significantly. Under varying initial densities, automata may fail to develop distinctive features sufficient for rule discrimination within smaller time steps *t*, leading to classification challenges for the model. For instance, rule 18, under a low initial density such as 0.2 and at small time steps, exhibits minimal density and a homogeneous state, making it difficult to distinguish between them. Expanding the range of initial densities of training sets is necessary to enhance the robustness of the CNN.

A uniform distribution of initial densities between 0.2 and 0.8 is selected. To ensure a sufficient training set, 6000 configurations are taken for each rule, and 600 configurations are used for the test set. The accuracy of CNN in recognizing different Wolfram rules is 0.985, indicating that even under a wide range of initial densities, CNN maintains high recognition accuracy for diverse Wolfram rules.

As shown in Table 7 and Table 8, based on the provided classification report and confusion matrix, the CNN model demonstrates strong performance in classifying Wolfram rules under expanded initial density conditions (e.g., densities uniformly sampled from 0.2 to 0.8). The overall accuracy of 0.985 (98.5%) indicates high effectiveness, with precision, recall, and F1-score all at 0.985, suggesting balanced performance across classes. The confusion matrix reveals minimal errors, with only 85 misclassifications out of 6000 samples, with most errors concentrated in specific rule pairs, such as rule 16 being confused with rules 18 and 48, as seen in the 25 and 21 errors, respectively. This minor confusion may stem from similarities in dynamical behaviors under certain density conditions, but the model maintains robustness, as evidenced by perfect F1-scores for rules 150, 182, and 190.

The CNN achieves high reliability in distinguishing Wolfram rules despite variations in initial density, with accuracy exceeding 98.5%. The results validate the model’s generalization capability, though future work could focus on refining feature extraction for rules with lower recall (e.g., rule 16 at 0.917) to further reduce errors. This performance underscores the potential of CNNs for complex automata classification tasks.

**Figure 12:**
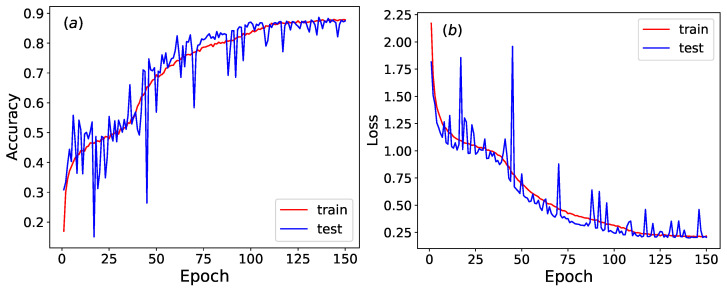
(**a**) The accuracy and (**b**) loss function of the trained neural network across epochs. The training and test sets undergo row-shuffling while sharing identical initial density conditions.

The training and test sets undergo row-shuffling while sharing identical initial density conditions. Row-shuffling is a control technique that randomly reorders the temporal sequence of rows in a Wolfram automata configuration. This process destroys dynamical patterns by disrupting the causal relationships between consecutive time steps, while preserving marginal statistics (e.g., density and local patterns) within each individual row. As illustrated in Figure 12, the accuracy and loss function of the trained neural network stabilize around epoch 150, and the accuracy and loss function fluctuate significantly.

As shown in Table 9 and Table 10, the provided confusion matrix and classification report evaluate the CNN model’s performance on classifying Wolfram rules with row-shuffled configuration. Row-shuffling disrupts the temporal sequence of Wolfram automata evolution, forcing the model to rely solely on static spatial features rather than time-dependent dynamics. The overall accuracy is 0.874 (87.4%), with a F1-score of 0.873, indicating a significant performance drop compared to scenarios with intact temporal data. The confusion matrix reveals systematic misclassifications: for instance, rule 18 is frequently confused with rule 6 (10 errors) and rule 22 (16 errors), while rule 150 shows low recall (0.600) due to 80 misclassifications as rule 90. This suggests that although row-shuffling preserves the marginal distribution of each row (such as density), density alone may be insufficient to distinguish all rules. In summary, while the model maintains moderate accuracy, the errors highlight the importance of temporal dynamics in Wolfram rule classification, and row-shuffling exposes limitations in feature learning.

**Figure 13:**
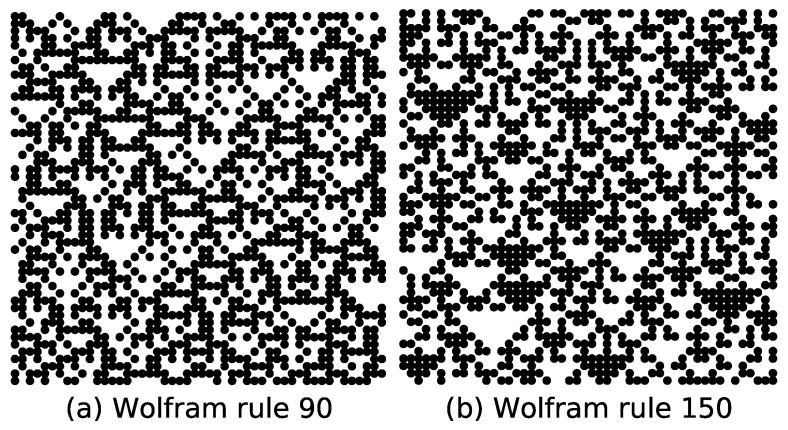
Configurations of rules 90 and 150 under random initial conditions.

Although rules 90 and 150 share the same asymptotic density ($\rho_{\infty }$ = 0.5) under random initial states, their configurations exhibit high visual similarity, making manual distinction challenging, as shown in Figure 13. However, a trained CNN achieves high classification accuracy in reliably identifying these rules. Although supervised learning provides prior knowledge of Wolfram rules, the neural network model can rapidly distinguish large volumes of Wolfram rule configurations with minimal training time—demonstrating a key advantage of machine learning for complex spatial–temporal pattern recognition tasks.

We employ a CNN as a supervised learning framework, due to its high effectiveness in image-like classification tasks, which offers superior efficiency and accuracy over traditional methods. By utilizing only a two-layer CNN architecture rather than deeper networks, we achieve optimal performance for the Wolfram automata configurations with small system dimensions. This design minimizes trainable parameters, reduces training time, and maintains high classification accuracy.

The CNN effectively identified unique spatiotemporal patterns and structural correlations within evolved configurations—even among rules sharing identical asymptotic densities, such as rules 90 and 150 (both with $\rho_{\infty }$ = 0.5). These discriminative features include propagation dynamics of active sites, neighborhood interaction patterns, and emergent fractal geometries, forming unique “ dynamical signatures” for each rule. This demonstrates that supervised learning can extract physically meaningful patterns beyond scalar metrics like density, enabling accurate classification based on structural evolution.

## 5. Unsupervised Learning of the Wolfram Automata

Unsupervised learning operates on unlabeled data, enabling autonomous discovery of latent structures and classifications without human guidance—particularly valuable when labeled data is limited or costly to acquire. This section will apply two prominent unsupervised techniques, namely autoencoders and principal component analysis (PCA), to study Wolfram automata. Outputs are dimensionally reduced to two- and one-dimensional spaces, prioritizing maximal physical relevance, a conclusion directly supported by evidence in this section.

### 5.1. Autoencoder Results of the Wolfram Automata

**Figure 14:**
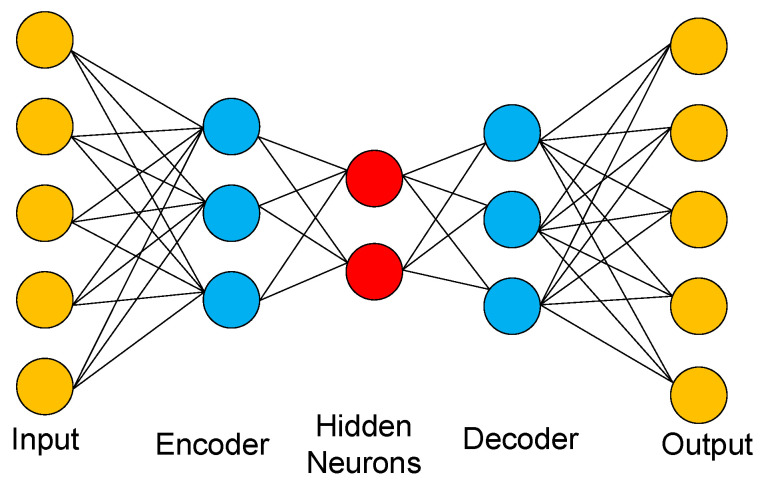
Schematic diagram of the autoencoder architecture. The fully connected autoencoder structure comprises an input layer, an encoder with four neural layers, a latent layer containing one or two neurons, a decoder with five neural layers, and an output layer. The neuron counts per layer follow the sequence: (256, 128, 64, 16, 2/1, 16, 64, 128, 256, 640), with ReLU activation functions applied throughout. Mean squared error (MSE) is selected as the loss function, and optimizers are assigned based on the number of neurons in latent layers: Adam (Adaptive Moment Estimation) for layers containing exactly two neurons, and stochastic gradient descent (SGD) for those with one neuron.

Autoencoders learn compressed representations of input data to reconstruct inputs and generate new samples resembling the original data distribution [38,39,40]. As depicted in Figure 14, the fully connected autoencoder architecture implemented in our study comprises an input layer, an encoder, a latent layer containing hidden neurons, a decoder, and an output layer. The inputs for the autoencoders are just the raw configurations of Wolfram automata. The Autoencoder employs the mean squared error (MSE) as its loss function; its core objective is to minimize the discrepancy between input data and reconstructed data, thereby learning an effective low-dimensional representation (latent representation) of the data. Through this compression–decompression mechanism, the latent layer generates a compressed representation that retains essential input features, enabling the decoder to reconstruct the original data patterns by reversing the dimensionality reduction process.

First, we select four Wolfram rules: 18, 90, 182, and 190. For these Wolfram rules, simulations are run on arrays of size L=30 and t=150, with the configurations from the last 50 time steps (Δt=50) being taken. For each rule, 2000 configurations are generated for the training set, and another 1000 configurations for the test set. By constraining the autoencoder’s output to two neurons, the configurations of Wolfram automata are compressed into two dimensions. After training is completed, each input $x_{i}$ from the test set produces a point $\left(h_{i1},h_{i2}\right)$ on the two-dimensional plane. For the data selection, we used the raw configurations of Wolfram automata and their row-shuffled counterparts, respectively.

**Figure 15:**
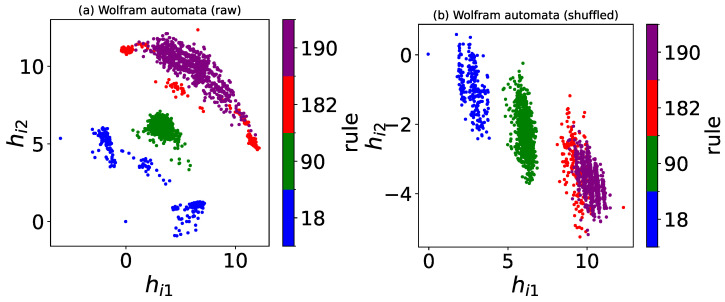
The autoencoder’s encoder, which comprises two hidden layers, projects both (**a**) raw configurations and (**b**) row-shuffled configurations, generated from rules 18, 90, 182, and 190, into a two-dimensional latent space. Different Wolfram rules are distinguished by a color map in the visualization.

As illustrated in Figure 15, the autoencoder compresses the raw and row-shuffled configurations of Wolfram automata into a two-dimensional latent space, respectively. The autoencoder separates the raw and row-shuffled configurations of different rules, despite partial overlap between the configurations of rules 182 and 190. Moreover, configurations of identical rules are clustered together, indicating that the autoencoder primarily relies on density to distinguish different Wolfram rules.

We also examine rules 34, 90, and 204, with raw configuration data sampled under identical conditions to those used for the aforementioned rules. By constraining the hidden layer to a single neuron, we investigate whether the features learned by the autoencoder correspond to the density of the Wolfram automata.

**Figure 16:**
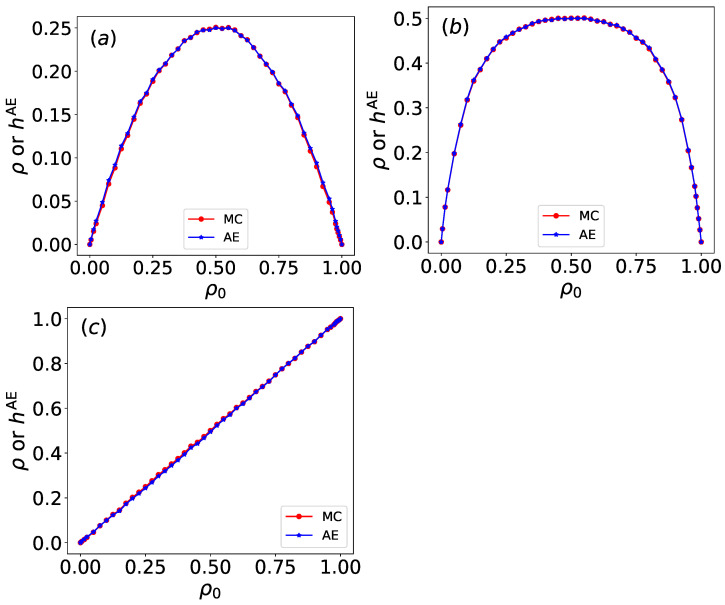
The raw configurations of Wolfram automata are encoded on a latent variable of a single hidden neuron as a function of the initial density $\rho_{0}$ for (**a**) rule 34, (**b**) rule 90, and (**c**) rule 204. The single-variable outputs (blue) of Wolfram automata autoencoder results are normalized, which are compared to the density ρ from the Monte Carlo simulations. Normalized latent outputs (blue) exhibit quantitative agreement with the density (red) derived from Monte Carlo simulations.

As shown in Figure 16, the regularized output $h^{\mathrm{AE}}$ for rules 34, 90, and 204 varies as a function of the initial density $\rho_{0}$. The deviations between $h^{\mathrm{AE}}$ and ρ are calculated using the mean absolute error (MAE) and mean squared error (MSE) methods. The MAE values for the Wolfram automata are 0.061 for rule 34, 0.078 for rule 90, and 0.0076 for rule 204. The MSE values for the Wolfram automata are 0.0057 for rule 34, 0.008 for rule 90, and 0.001 for rule 204. $h^{\mathrm{AE}}$ and ρ are in close agreement. Mutual information (MI) measures the degree of dependency between variables, capturing both linear and nonlinear associations. The calculated MI values are 2.06 for rule 34, 1.77 for rule 90, and 3.09 for rule 204. High mutual information is found between $h^{\mathrm{AE}}$ and ρ, indicating that the autoencoder’s latent variable successfully captures the density information. Therefore, the regularized single latent variable can be interpreted as the density.

### 5.2. PCA Results of Wolfram Automata

Principal component analysis (PCA) [41,42,43], as an unsupervised learning algorithm, reduces data dimensionality through orthogonal transformation. It identifies orthogonal directions of maximum variance in the data space, converting potentially correlated variables into linearly uncorrelated principal components (PCs). For Wolfram automata analysis, we focus exclusively on the first two principal components, which capture the dominant sources of variation.

This process can be conceptualized as projecting high-dimensional data onto a lower-dimensional manifold—specifically, selecting projection axes that maximize retained variance. Such projections preserve maximal information integrity after dimensionality reduction. In alignment with prior methodologies, we prioritize reductions to a physically interpretable low-dimensional space.

The PCA methodology is implemented through the following specific procedure. Simulations are run on arrays of size L=30 with temporal evolution spanning t=150 steps. From each simulation, configurations are extracted exclusively from the last 50 time steps (Δt=50). These configurations are structured into *M*-dimensional vectors $x_{i}$, where M=L×Δt. For each distinct rule, 1000 such configurations are generated, resulting in a comprehensive dataset of N=1000 samples. The full dataset is organized into an (N×M)-dimensional matrix $X={\left(x_{1},x_{2},\ldots ,x_{i},\ldots ,x_{N}\right)}^{T}$, which serves as the foundational input for PCA computations.

PCA operates by determining principal components through a linear transformation Y=XW. Here, the transformation matrix $W=(w_{1},w_{2},\ldots ,w_{K})$ has dimensions (M×K), with each column vector $w_{n}$ representing a weighted component of dimension *M*. When K≪M, this transformation achieves significant dimensionality reduction.

The principal component (PC) directions are derived by analyzing the real symmetric covariance matrix $X^{T}X$, which has dimensions (M×M). For the case K=M, the directions $w_{n}$ correspond precisely to the eigenvectors of $X^{T}X$, satisfying the eigenvalue equation:

(5) $$X^{T}Xw_{n}=\lambda_{n}w_{n}\;.$$

The eigenvalues $\lambda_{n}$ are sorted in descending order ($\lambda_{1}\geq \lambda_{2}\geq \cdots \geq \lambda_{M}\geq 0$), quantifying the variance of X along each eigenvector direction. Dimensionality reduction is subsequently achieved by retaining only the first few eigenvectors $w_{n}$ associated with the largest $\lambda_{n}$. In standard PCA terminology, the normalized eigenvalues ${\tilde{\lambda }}_{n}=\lambda_{n}/\sum_{i=1}^{M}\lambda_{i}$ define the **explained variance ratio**. This ratio represents the proportion of total variance captured by the *n*-th principal component, thereby quantifying its statistical significance in the reduced-dimensional representation.

**Figure 17:**
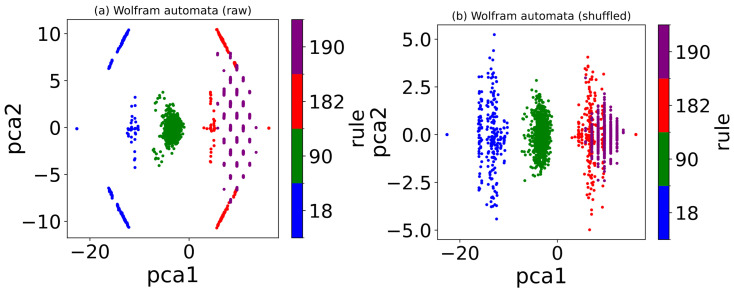
PCA retains two principal components to compress both (**a**) raw and (**b**) row-shuffled configurations of rules 18, 90, 182, and 190 into two dimensions. A color map distinguishes different Wolfram rules in the visualization.

For rules 18, 90, 182, and 190, PCA projects the raw and row-shuffled configurations of Wolfram automata into two dimensions by retaining two principal components, respectively, as illustrated in Figure 17. Similar to autoencoder results, PCA also separates raw and row-shuffled configurations of different Wolfram rules into distinct clusters. This indicates that the PCA primarily relies on density to distinguish different Wolfram rules as well.

**Figure 18:**
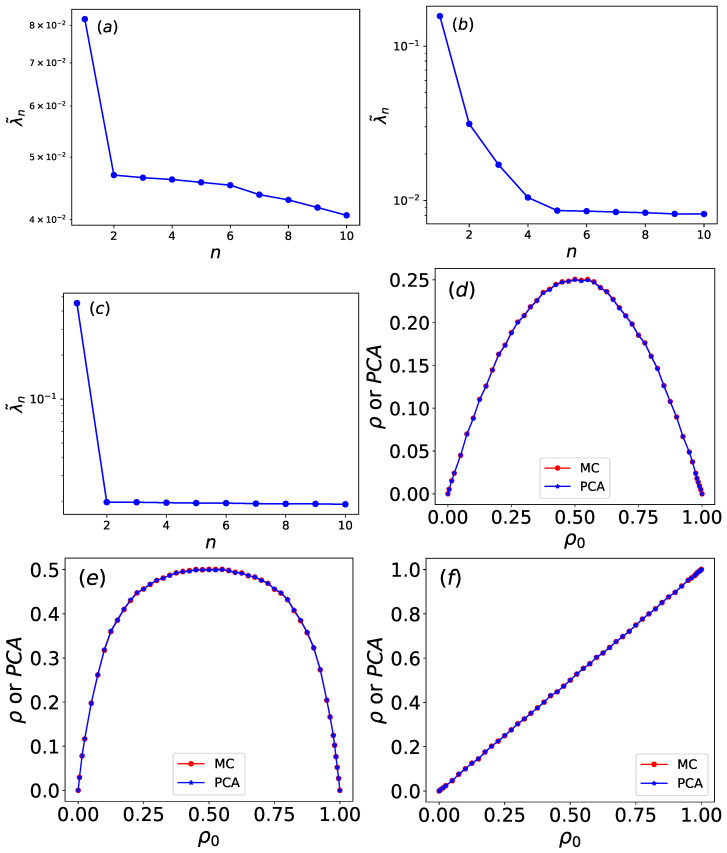
PCA explained variance ratios of the first 10 principal components for (**a**) rule 34, (**b**) rule 90, and (**c**) rule 204. PCA results for (**d**) rule 34, (**e**) rule 90, and (**f**) rule 204. The normalized first principal component (blue) varies as a function of the initial density $\rho_{0}$, which is compared to the density (red) from the Monte Carlo simulations.

As shown in Figure 18a,b, the variance ratios of the first and second principal components (PC1 and PC2) differ significantly across rules. For rule 34, the variance ratios are 0.0818 (PC1) and 0.04 (PC2), while for rule 90, they are 0.156 (PC1) and 0.0313 (PC2). In both cases, PC1 contributes the most to the total variance, yet its ratio remains relatively small. The variance-explained ratio of the PC1 and PC2 is generally higher for linear data, essentially because the linear nature of PCA aligns well with the inherent structure of linear data. The density ρ of rule 34 is a quadratic function of the initial density $\rho_{0}$, indicating strong nonlinearity in its dynamics. For nonlinear data, however, the linear assumption of PCA limits its ability to capture major variations, resulting in a more dispersed and lower variance explained ratio. This explains the suppressed variance ratio of PC1 (0.0818) and the similar ratio (0.04) for subsequent components in rule 34. For rule 90, although ρ also exhibits nonlinear dependence on $\rho_{0}$, this relationship is weaker. Thus, PCA more effectively concentrates variance into PC1, yielding a higher ratio (0.156). The residual nonlinearity distributes into minor PCs (e.g., PC2 ratio = 0.0313), consistent with PCA’s linearity constraints.

In contrast, as shown in Figure 18c, the variance ratio of PC1 for rule 204 is 0.45, which is substantially higher than those of rules 34 and 90. Since the density of rule 204 maintains a linear relationship with the initial density, the variance is more effectively captured by the first principal component.

Furthermore, Figure 18d–f compare the normalized first principal component with the density for rules 34, 90, and 204. The deviations between normalized PC1 and ρ are also calculated using MAE and MSE methods. The MAE values for the Wolfram automata are 0.059 for rule 34, 0.054 for rule 90, and 0.00016 for rule 204. The MSE values are 0.0053 for rule 34, 0.0038 for rule 90, and 5.3 ×$10^{-8}$ for rule 204. PC1 and ρ show a high degree of agreement. The calculated MI values are 2.302 for rule 34, 3.71 for rule 90, and 3.35 for rule 204. Therefore, the normalized first principal component can be interpreted as the density.

Both the regularized single latent variable $h^{\mathrm{AE}}$ of the autoencoder and the normalized first principal component (PC1) of PCA show high consistency with the density. However, quantitative comparisons based on MAE, MSE, and MI reveal that PC1 exhibits smaller deviations. This superior alignment is achieved with significantly greater computational efficiency, as PCA reduces the feature space in under one second, whereas the autoencoder requires approximately two minutes of training.

Unsupervised learning methods, including autoencoder and PCA, were employed to reduce dimensionality and cluster raw and row-shuffled configurations of Wolfram automata. The two-dimensional projections from both methods separated the raw and row-shuffled configurations of different Wolfram rules into distinct clusters, indicating that autoencoder and PCA primarily relies on density to distinguish between these rules. When constrained to one latent dimension, both methods consistently captured the density (a core dynamical property), demonstrating close alignment with Monte Carlo simulation results across multiple rules. This indicates that despite their simplicity, these unsupervised methods are capable of extracting the most dominant and physically interpretable variable in the system.

## 6. Summary

In this paper, we applied numerical simulations, computation methods, and supervised and unsupervised learning methods to study the asymptotic density and dynamical evolution mechanisms of the (1+1)-dimensional Wolfram automata. Through numerical simulations, we extended beyond single-site initial states to specific sequences, including rule 146 with “…0, 1, 1, 0, 1, 1, 0, 1, 1, 1, 0, 1, 1, 0, 1, 1…” and rule 154 with “…0, 1, 0, 1, 0, 1, 1, 0, 1, 0, 1…”. Rules 26, 82, 146, 154, and 210 also evolve into Sierpiński triangle patterns over time.

The asymptotic density of Wolfram rules was numerically determined. For complex rules such as rules 18 and 90, the asymptotic density $\rho_{\infty }$ converges to a well-defined value under most disordered initial configurations with initial density $\rho_{0}$. However, under extremely low or high initial densities (e.g., $\rho_{0}\leq 0.01$ or $\rho_{0}\geq 0.99$), like *L* = 2000, even after extended evolution (e.g., *t* = $10^{6}$), $\rho_{t}$ have a substantial deviation from $\rho_{\infty }$. This discrepancy may stem from finite-size effects; as the system size increases, $\rho_{t}$ will approach the asymptotic density $\rho_{\infty }$ more closely. The density ρ of simple Wolfram rules stabilizes within a small number of time steps, with the asymptotic density $\rho_{\infty }$ depending on the initial density $\rho_{0}$. Certain rules, such as rules 2, 4, and 16, or rules 34 and 48, rely on different local update conditions. Yet, under identical initial density, even though their configurations evolve through different pathways over time, they converge to the same asymptotic density, suggesting that they share equivalent underlying dynamical mechanisms.

With supervised learning, a trained CNN achieves high accuracy in identifying these rules. Although supervised learning provides prior knowledge of Wolfram rules, the neural network model can rapidly distinguish large volumes of Wolfram rule configurations with minimal training time—demonstrating a key advantage of machine learning for complex spatial–temporal pattern recognition tasks.

The unsupervised learning methods, PCA and autoencoders, were also employed. The two-dimensional projections from both methods separated the raw and row-shuffled configurations of different Wolfram rules into distinct clusters, indicating that autoencoders and PCA primarily rely on density to distinguish between these rules. Once the output is restricted to one dimension, both methods yield an output that shows strong agreement with the density. Due to its simpler learning mechanism, PCA achieves shorter computation time and greater operational efficiency compared to the autoencoder.

For future research directions, our study can be extended in several meaningful ways. First, our focus on (1+1)-dimensional Wolfram automata leaves open questions about higher-dimensional or stochastic systems, which we plan to explore in future work. Second, although our machine learning models achieve high accuracy, interpretability remains a challenge; we will investigate explainable AI techniques to bridge model decisions with physical insights. Finally, computational costs for large-scale automata necessitate optimizations like distributed computing. Addressing these aspects will extend the framework’s applicability to broader complex systems.

## Figures and Tables

**Table 1:** Boolean functions of Wolfram automata.

	$s_{i}$ (*t* + 1)		$s_{i}$ (*t* + 1)
rule 1	$\neg (s_{i-1}\left(t\right)\vee s_{i}\left(t\right)\vee s_{i+1}\left(t\right))$	rule 2	$s_{i+1}\left(t\right)\wedge \left(\neg \left(s_{i-1}\left(t\right)\vee s_{i}\left(t\right)\right)\right)$
rule 4	$s_{i}\left(t\right)\wedge \left(\neg \left(s_{i-1}\left(t\right)\vee s_{i+1}\left(t\right)\right)\right)$	rule 16	$s_{i-1}\left(t\right)\wedge \left(\neg \left(s_{i}\left(t\right)\vee s_{i+1}\left(t\right)\right)\right)$
rule 18	$\neg s_{i}\left(t\right)\wedge \left(s_{i-1}\left(t\right)⊕s_{i+1}\left(t\right)\right)$	rule 30	$s_{i-1}\left(t\right)⊕\left(s_{i}\left(t\right)\vee s_{i+1}\left(t\right)\right)$
rule 34	$s_{i+1}\left(t\right)\wedge \left(\neg s_{i}\left(t\right)\right)$	rule 36	$\left(s_{i-1}\left(t\right)⊕s_{i}\left(t\right)\right)\wedge \left(s_{i}\left(t\right)⊕s_{i+1}\left(t\right)\right)$
rule 48	$s_{i-1}\left(t\right)\wedge \left(\neg s_{i}\left(t\right)\right)$	rule 90	$s_{i-1}\left(t\right)⊕s_{i+1}\left(t\right)$
rule 110	$\left(s_{i}\left(t\right)\wedge \left(\neg s_{i-1}\left(t\right)\right)\right)\vee \left(s_{i}\left(t\right)⊕s_{i+1}\left(t\right)\right)$	rule 170	$s_{i+1}\left(t\right)$
rule 204	$s_{i}\left(t\right)$	rule 240	$s_{i-1}\left(t\right)$

**Table 2:** The Fitted density $\rho_{Fitted}$ of rules 1, 2, 4, 16, 34, 48, 36, 170, 204, and 240, and approximate asymptotic density $\rho_{\infty }$.

	$\rho_{\mathrm{Fitted}}$	$\rho_{\infty }$
rule 1	$0.99\rho_{0}^{4}-2.99\rho_{0}^{3}+2.99\rho_{0}^{2}-1.01\rho_{0}+0.499$	$\rho_{0}^{4}-3\rho_{0}^{3}+3\rho_{0}^{2}-p+0.5$
rules 2, 4, and 16	$0.99\rho_{0}^{3}-1.99\rho_{0}^{2}+1.01\rho_{0}-3.02\times 10^{-5}$	$\rho_{0}^{3}-2\rho_{0}^{2}+\rho_{0}$
rules 34 and 48	$-0.99\rho_{0}^{2}+0.99\rho_{0}-5.2\times 10^{-5}$	$-\rho_{0}^{2}+\rho_{0}$
rule 36	$-3.01\rho_{0}^{4}+6.02\rho_{0}^{3}-4.01\rho_{0}^{2}+1.02\rho_{0}$	$-3\rho_{0}^{4}+6\rho_{0}^{3}-4\rho_{0}^{2}+\rho_{0}$
rules 170, 204, and 240	$\rho_{0}$	$\rho_{0}$

**Table 3:** Confusion matrix for CNN-based classification of Wolfram rules under identical initial density conditions in training and test sets.

	6	16	18	22	36	48	90	150	182	190
6	200	0	0	0	0	0	0	0	0	0
16	0	200	0	0	0	0	0	0	0	0
18	0	0	199	0	1	0	0	0	0	0
22	0	0	0	200	0	0	0	0	0	0
36	0	0	0	0	200	0	0	0	0	0
48	0	2	0	0	0	198	0	0	0	0
90	0	0	0	0	0	0	200	0	0	0
150	0	0	0	0	0	0	0	200	0	0
182	0	0	0	0	0	0	0	0	200	0
190	0	0	0	0	0	0	0	0	0	200

**Table 4:** Classification report of CNN of Wolfram rules under identical initial density conditions in training and test sets.

Class	Precision	Recall	F1-Score
6	1.000	1.000	1.000
16	0.990	1.000	0.995
18	1.000	0.995	0.997
22	1.000	1.000	1.000
36	0.995	1.000	0.998
48	1.000	0.990	0.995
90	1.000	1.000	1.000
150	1.000	1.000	1.000
182	1.000	1.000	1.000
190	1.000	1.000	1.000

**Table 5:** Confusion matrix of CNN of Wolfram rules under different initial density conditions in training and test sets.

	6	16	18	22	36	48	90	150	182	190
6	190	0	10	0	0	0	0	0	0	0
16	0	138	41	0	2	19	0	0	0	0
18	0	0	199	0	1	0	0	0	0	0
22	0	0	8	192	0	0	0	0	0	0
36	63	0	7	0	130	0	0	0	0	0
48	0	31	3	0	0	166	0	0	0	0
90	1	0	8	2	0	0	188	1	0	0
150	0	0	0	6	1	0	1	190	0	2
182	0	0	0	0	0	0	0	0	200	0
190	0	0	0	0	0	0	0	0	0	200

**Table 6:** Classification report of CNN of Wolfram rules under different initial density conditions in training and test sets.

Class	Precision	Recall	F1-Score
6	0.748	0.950	0.837
16	0.817	0.690	0.748
18	0.721	0.995	0.836
22	0.960	0.960	0.960
36	0.970	0.650	0.778
48	0.897	0.830	0.862
90	0.995	0.940	0.967
150	0.995	0.950	0.972
182	1.000	1.000	1.000
190	0.990	1.000	0.995

**Table 7:** Confusion matrix for CNN classification with expanded initial density range.

	6	16	18	22	36	48	90	150	182	190
6	595	0	4	1	0	0	0	0	0	0
16	3	550	25	0	1	21	0	0	0	0
18	5	2	590	0	0	0	3	0	0	0
22	2	0	8	590	0	0	0	0	0	0
36	0	0	0	0	600	0	0	0	0	0
48	0	16	0	0	0	584	0	0	0	0
90	0	0	0	0	0	0	600	0	0	0
150	0	0	0	0	0	0	0	600	0	0
182	0	0	0	0	0	0	0	0	600	0
190	0	0	0	0	0	0	0	0	0	600

**Table 8:** Classification report for CNN with expanded initial density range.

Class	Precision	Recall	F1-Score
6	0.983	0.992	0.988
16	0.968	0.917	0.942
18	0.941	0.983	0.962
22	0.998	0.983	0.991
36	0.998	1.000	0.999
48	0.965	0.973	0.969
90	0.995	1.000	0.998
150	1.000	1.000	1.000
182	1.000	1.000	1.000
190	1.000	1.000	1.000

**Table 9:** Confusion matrix for CNN with row-shuffled configuration (identical initial density).

	6	16	18	22	36	48	90	150	182	190
6	185	0	12	1	0	0	2	0	0	0
16	0	183	0	0	0	17	0	0	0	0
18	10	0	174	0	16	0	0	0	0	0
22	0	0	30	170	0	0	0	0	0	0
36	0	0	0	0	200	0	0	0	0	0
48	1	27	23	0	0	149	0	0	0	0
90	0	0	0	0	0	0	183	17	0	0
150	0	0	0	0	0	0	80	120	0	0
182	0	0	0	0	0	0	0	0	200	0
190	0	0	0	0	0	0	0	0	16	184

**Table 10:** Classification report for CNN with row-shuffled configuration (identical initial density).

Class	Precision	Recall	F1-Score
6	0.944	0.925	0.934
16	0.871	0.915	0.893
18	0.728	0.870	0.793
22	0.994	0.850	0.916
36	0.926	1.000	0.962
48	0.898	0.745	0.814
90	0.691	0.915	0.787
150	0.876	0.600	0.712
182	0.926	1.000	0.962
190	1.000	0.920	0.958

## Data Availability

The original contributions presented in this study are included in the article. Further inquiries can be directed to the corresponding author.
